# Adult Body Height Is a Good Predictor of Different Dimensions of Cognitive Function in Aged Individuals: A Cross-Sectional Study

**DOI:** 10.3389/fnagi.2016.00217

**Published:** 2016-09-16

**Authors:** Vitor H. Pereira, Patrício S. Costa, Nadine C. Santos, Pedro G. Cunha, Margarida Correia-Neves, Joana A. Palha, Nuno Sousa

**Affiliations:** ^1^Life and Health Sciences Research Institute, (ICVS) School of Health Sciences, University of MinhoBraga, Portugal; ^2^ICVS/3B's, PT Government Associate LaboratoryBraga/Guimarães, Portugal; ^3^Centro Hospitalar do Alto Ave – EPEGuimarães, Portugal; ^4^Clinical Academic CenterBraga, Portugal

**Keywords:** height, weight, cognition, mood, aging, community

## Abstract

**Background:** Adult height, weight, and adiposity measures have been suggested by some studies to be predictors of depression, cognitive impairment, and dementia. However, the presence of confounding factors and the lack of a thorough neuropsychological evaluation in many of these studies have precluded a definitive conclusion about the influence of anthropometric measures in cognition and depression. In this study we aimed to assess the value of height, weight, and abdominal perimeter to predict cognitive impairment and depressive symptoms in aged individuals.

**Methods and Findings:** Cross-sectional study performed between 2010 and 2012 in the Portuguese general community. A total of 1050 participants were included in the study and randomly selected from local area health authority registries. The cohort was representative of the general Portuguese population with respect to age (above 50 years of age) and gender. Cognitive function was assessed using a battery of tests grouped in two dimensions: general executive function and memory. Two-step hierarchical multiple linear regression models were conducted to determine the predictive value of anthropometric measures in cognitive performance and mood before and after correction for possible confounding factors (gender, age, school years, physical activity, alcohol consumption, and smoking habits). We found single associations of weight, height, body mass index, abdominal perimeter, and age with executive function, memory and depressive symptoms. However, when included in a predictive model adjusted for gender, age, school years, and lifestyle factors only height prevailed as a significant predictor of general executive function (β = 0.139; *p* < 0.001) and memory (β = 0.099; *p* < 0.05). No relation was found between mood and any of the anthropometric measures studied.

**Conclusions and Relevance:** Height is an independent predictor of cognitive function in late-life and its effects on the general and executive function and memory are independent of age, weight, education level, gender, and lifestyle factors. Altogether, our data suggests that modulators of adult height during childhood may irreversibly contribute to cognitive function in adult life and that height should be used in models to predict cognitive performance.

## Introduction

Cognitive impairment and depressive symptoms have a significant impact on the quality of life of the elderly (Santos et al., 2013; Meeks et al., 2016). A comprehensive knowledge of the determinants of both mood and cognition during aging is imperative to plan interventions aimed to ameliorate cognitive decline and depressive symptoms in late life.

Depressive symptoms and cognitive impairment often co-occur and are influenced by multiple factors (Santos et al., 2013, 2014). Together with age, low educational level is widely recognized as one of the main predictors of cognitive decline and an important risk factor for dementia (Santos et al., 2013, 2014; Xu et al., 2016). In addition, cognition is also influenced by lifestyle behaviors (Santos et al., 2014). Regular physical activity and moderate alcohol consumption are associated with better cognitive performance (Santos et al., 2014). On the other side, the best predictor of mood is gender; in fact, women consistently display more depressive symptoms than men during life span (Santos et al., 2013, 2014). The influence of lifestyle in mood is more controversial and varies among cohorts.

Given the exceedingly high incidence of obesity and diabetes worldwide several studies have also explored possible associations between metabolic parameters, cognition and mood. The abdominal perimeter and body mass index (BMI, defined as the weight in kilograms divided by the square of the height in meters) were shown to be associated with cognitive impairment (Sabia et al., 2009) and depressive symptoms (Santos et al., 2014). In one study, individuals with higher BMI performed worst in neurocognitive tests independently of the presence of risk factors such as diabetes or dyslipidemia (Cournot et al., 2006). However, these data are not reproducibly among different cohorts and even contradictory findings have been reported (Santos et al., 2014; Smith et al., 2014; Yesavage et al., 2014). This raises the question about the true role of anthropometric parameters to predict both mood and cognition.

In this context, it is interesting to note that some studies revealed that height itself is a predictor of cognition. Adults with short stature display worst cognitive performance, attain lower educational levels in school, and present higher risk of early onset dementia and dementia-related death (Magnusson et al., 2006; Case and Paxson, 2008; Zhang et al., 2009; Nordström et al., 2013; Guven and Lee, 2014; Russ et al., 2014). These associations were observed in different regions of the globe including Europe (Guven and Lee, 2014), United States (Case and Paxson, 2008), and Caribbean countries (Maurer, 2010); in different ages (Quan et al., 2013; Stewart et al., 2015); and in specific sub-groups of individuals such as patients with type II diabetes or atherosclerotic disease (Weinstein et al., 2013; West et al., 2015). Adult body height is highly influenced by childhood environment (comprehensive of socio-economic, nutrition and health conditions) and early-life events (Guven and Lee, 2013). The adversities experienced during these periods are known to be important determinants of disease in adult life and may explain the association between cognition and height (Guven and Lee, 2014).

In this sense, the main goal of this project was to clarify the value of weight, height, and abdominal perimeter to predict cognitive performance (memory and executive function) and depressive symptoms in adult life. This analysis was controlled for factors previously shown to influence cognition and mood such as age, gender, alcohol consumption, physical activity, and smoking (Santos et al., 2014). The neuropsychological assessment was performed in a cohort of community-dwellers older than 50-years of age.

## Methods

### Ethics statement

The study was conducted in accordance with the Declaration of Helsinki (59th Amendment) and was approved by the national ethical committee (Comissão Nacional de Protecção de Dados) and by the local ethics review boards (Hospital de Braga, Braga; Centro Hospitalar do Alto Ave, Guimarães). The study goals and the neurocognitive/psychological and clinical assessments were explained to all participants, who all gave informed consent.

### Characterization of the cohort

The cohort was composed by 1051 participants randomly selected from two cities in the north of Portugal (Guimarγes and Vizela) using the local area health authority registries as described elsewhere (Santos et al., 2013, 2014). The cohort is representative of the Portuguese population with respect to gender (females, *n* = 560; 53.3%) and age (range: 50–97 years; M = 67.2, SD = 9.24). All the participants were community-dwellers. The majority of them was retired (*n* = 763, females 51.8%) and located in the medium socio-economic stratum in the Graffar scale (Class III; 61.6%, females 47.3%). The percentage of the cohort that attended school for [1–2], [3–4], [5–8], [9–12], and ≥13 years was 9.8, 61.0, 6.9, 7.5, and 1.5%, respectively. On socio-demographic measures, Portugal ranks close to the OECD (Organization for Economic Co-operation and Development; www.oecd.org/) average. These data are detailed in Table 1.

**Table 1:** **Socio-demographic characterization of the cohort**.

**Variable**	***n***	**%**
**GENDER**		
Female	560	53.3%
Male	491	46.7%
**GRAFFAR CLASS**		
Class I	3	1.1%
Class II	10	3.7%
Class III	167	61.6%
Class IV	91	33.6%
**SCHOLARITY**		
None	140	13.3%
1–2	103	9.8%
3–4	641	61.0%
5–8	72	6.9%
9–12	79	7.5%
13+	16	1.5%
**SMOKING STATUS**		
Non-smoker	735	70.5%
Former smoker	232	22.3%
Smoker	75	7.2%
**ALCOHOL CONSUMPTION**		
None	303	29.4%
<50 g/day	482	46.8%
>50 g/day	246	23.9%
**PHYSICAL ACTIVITY**		
None	670	64.3%
<3 times a week	154	14.8%
>3 times a week	218	20.9%
Age (years)	67·2	*SD* = 9.2
Height (m)	1·60	*SD* = 0·09
Weight (kg)	72·23	*SD* = 12.36
BMI (kg/m^2^)	28·41	*SD* = 4.63
Abdominal Perimeter (cm)	98·85	*SD* = 10.54

SD, standard deviation.

### Physical and lifestyle variables

Physical measures included weight (Kg), height (m), and abdominal perimeter (cm). For lifestyle, alcohol consumption (none, 50 or less, and more than 50 grams/day), physical activity status (none, <3, and over 3 times per week), and smoking habits (non-smoker, former smoker, and current smoker) were considered. Alcohol consumption was calculated and recorded as total grams/day, taking as reference the estimations of grams of alcohol per glass for each consumed beverage. Physical activity included any planned activities (e.g., walking, jogging, swimming) that comprised a continuous 30 min effort (which could range from light, to moderate and vigorous) above the everyday living activities such as the case of regular short walk to the grocery store. Activity quantity rather than intensity was considered due to the mixed clinical profiles and age range of the study population. Alcohol consumption and smoking habits consumption were self-reported by the participants during the clinical interview, and were referent to the current habits.

### Neuropsychological/cognitive evaluation

Tests were selected to provide cognitive profiles (general cognitive status and executive and memory functions). A team of trained psychologists conducted the neurocognitive/ psychological evaluations as described elsewhere (Santos et al., 2015). The evaluation included the following instruments: the Mini Mental State Examination (MMSE; Folstein et al., 1975), which is the most widely used cognitive mental status-screening test and assesses orientation, word recall, attention and calculation, language and visual-construction abilities; the Digit Span Test (Della Sala et al., 1995), used as a measure of short-term memory, working memory, and attention; the Stroop Test to evaluate the ability to resist to interference and to assess cognitive flexibility and inhibitory control; the Selective Reminding Test (SRT), to evaluate verbal learning and memory through the parameters long-term storage (LTS), consistent-term retrieval (CLTR), and delayed recall (Buschke et al., 1995); and the Controlled Oral Word Association Test (COWAT-FAS), which is a measure of verbal fluency. All neurocognitive test scores were converted into *z* scores to express all variables in the same scale and then grouped in two different categories: general and executive function (GENEXEC) and memory (MEM) based on a factorial analysis as described elsewhere (Santos et al., 2015). General and executive function was composed of the parameters MMSE, Stroop (parameters: words, colors, and words/colors), FAS (parameter: admissible), and digits (parameter: backward); and the memory function composed of the SRT test variables (parameters: CLTR, LTS, and delayed recall). Participants who met the established MMSE threshold criteria for dementia (*n* = 51) or were unable/unwilling to complete the test (*n* = 7) were excluded from further analysis. The Geriatric Depression Scale (GDS, long-version) was used to assess depressive mood, which was considered a single dimension.

### Statistical analysis

Pearson correlations were calculated to measure the strength of the association between the studied quantitative variables. Afterwards, a Multiple Linear Regression Model (MLRM) was performed for each of the composite scores previously calculated (GDS; GENEXEC, MEM) using two blocks of variables as predictors. In block 1 we aimed to understand the value of height, weight, and abdominal perimeter to predict cognition and depression and therefore, these parameters were included in the model together with gender and age. In the second block possible confounding variables previously shown to be involved in cognition such as school years, smoking status, alcohol consumption, and physical activity, were added to the model. Besides regression coefficients (and confidence intervals), betas and measures of model fit (*R*^2^, $R_{\text{adjusted},}^{2}$ and Δ*R*^2^) are also presented. All the statistical analyses were conducted using the software SPSS 22.0 (IBM, USA).

## Results

Table 2 shows the Pearson correlation coefficients (and sample size) for age, weight, height, BMI, cognition, and mood dimensions. We found significant correlations between anthropometric measures and general executive performance (GENEXEC), memory function (MEM), and mood (GDS). Height was positively correlated with GENEXEC (*r* = 0.337; *p* < 0.01) and MEM (*r* = 0.187; *p* < 0.01) and, negatively correlated with GDS (*r* = −0.269, *p* < 0.01). This means that superior height values are associated with better executive performance, better memory, and less depressive symptoms. Weight was also significantly and positively correlated with GENEXEC (*r* = 0.082; *p* < 0.01) and negatively correlated with GDS (*r* = −0.074; *p* < 0.05) meaning that higher weight scores are associated with higher executive performance and better mood. Both BMI and abdominal perimeter were significantly and negatively correlated with GENEXEC (r_BMI_ = −0.143, *p* < 0.01; r_AP_ = −0.160, *p* < 0.01) and MEM (r_BMI_ = −0.143, *p* < 0.01; r_AP_ = −0.115, *p* < 0.01) and positively correlated with GDS (r_BMI_ = 0.105, *p* < 0.01; r_AP_ = 0.063, *p* < 0.05) meaning that higher adiposity, estimated by abdominal perimeter and BMI, is associated with lower performance on both cognitive dimensions and more depressive symptoms. Abdominal perimeter showed a strong positive correlation with weight and BMI and a less significant, but yet positive, correlation with height meaning that abdominal perimeter is higher in heavier and taller individuals. Older age was associated with worst performance in both cognitive dimensions (r_GENEXEC_ = −0.439, *p* < 0.01; and for r_MEM_ = −0.383, *p* < 0.01) and was not associated with depressive symptoms. Elder individuals also displayed higher abdominal perimeter (*r* = 0.204, *p* < 0.01) and shorter stature (*r* = −0.203, *p* < 0.01).

**Table 2:** **Pearson correlation coefficients (and sample size) for age, weight, height, body mass index, cognition, and mood dimensions**.

	**GENEXEC**	**MEM**	**GDS**	**Age**	**Weight**	**Height**	**BMI**
MEM	0.598^**^(1051)						
GDS	−0.301^**^(1044)	−0.238^**^(1044)					
Age	−0.439^**^(1051)	−0.383^**^(1051)	0.039(1044)				
Weight	0.082^**^(1007)	0.043(1007)	−0.074^*^(1000)	−0.076^*^(1007)			
Height	0.337^**^(1007)	0.187^**^(1007)	−0.269^**^(1000)	−0.203^**^(1007)	0.453^**^(1007)		
BMI	−0.143^**^(1007)	−0.082^**^(1007)	0.105^**^(1000)	0.058(1007)	0.761^**^(1007)	−0.215^**^(1007)	
Abdominal Perimeter	−0.160^**^(1004)	−0.115^**^(1004)	0.063^*^(997)	0.204^**^(1004)	0.777^**^(1004)	0.064^*^(1004)	0.790^**^(1004)

* p < 0.05;

** *p < 0.01*.

### Multiple linear regression models (MLRM)

Two-step hierarchical multiple linear regression models were conducted to determine the predictive value of anthropometric measures in cognitive performance and mood (depressive symptoms) before and after correction for possible confounding factors (gender, age, school years, and lifestyle factors—physical activity, alcohol consumption, and smoking habits).

Table 3 shows the results of the two-step hierarchical MLRM conducted to determine the predictive value of anthropometric measures in cognitive performance (general executive: GENEXEC; and memory: MEM) and mood (depressive symptoms: GDS) before and after controlling for possible confounding factors (gender, age, school years, and lifestyle factors—physical activity, alcohol consumption, and smoking habits). The first step models explained 13.5% of the variance of GDS [*F*_(5, 981)_ = 30.67; *p* < 0.001], 25.9% of the variance of GENEXEC [*F*_(5, 988)_ = 68.94; *p* < 0.001], and 17.1% of the variance of MEM [*F*_(5, 988)_ = 40.68; *p* < 0.001]. Concerning GDS, only gender was significantly correlated with depressive symptoms, which were more prevalent in women (β = −0.329; *p* < 0.01). Age, height, weight, and abdominal perimeters lost significance in predicting GDS when used in this model. For cognitive function, age and height were significant predictors of both GENEXEC and MEM. Specifically, older age was associated with a worst performance in both general and executive function (β = −0.345; *p* < 0.001) and memory (β = 0.236; *p* < 0.001). Higher height was associated with a better performance in both general executive function (β = −0.236; *p* < 0.001) and memory (β = 0.167; *p* < 0.001). Abdominal perimeter was negatively associated with general executive function (β = −0.143; *p* < 0.05). Interestingly, weight lost significance when used in this model.

**Table 3:** **Multiple linear regression models for variables predicting mood and cognitive performance dimensions**.

**Dependent Variables**	**GDS**			**GENEXEC**			**MEM**		
	**B [CI95%]**	**S.E**	**Beta**	**B [CI95%]**	**S.E**	**Beta**	**B [CI95%]**	**S.E**	**Beta**
Gender^a^	−0.66 [−0.829; −0.491]	0.086	−0.329^***^	0.059 [−0.096; 0.214]	0.079	0.029	−0.145 [−0.308; 0.018]	0.083	−0.073
Age	0.002 [−0.005; 0.009]	0.004	0.018	−0.038 [−0.044; −0.031]	0.003	−0.345^***^	−0.038 [−0.045; −0.03]	0.004	−0.345^***^
Height	−0.337 [−1.447; 0.772]	0.565	−0.029	2.716 [1.702; 3.731]	0.517	0.236^***^	1.916 [0.85; 2.982]	0.543	0.167^***^
Weight	−0.003 [−0.014; 0.007]	0.005	−0.038	0.004 [−0.005; 0.014]	0.005	0.053	0.001 [−0.009; 0.011]	0.005	0.013
Abdominal Perimeter	0.009 [−0.003; 0.02]	0.006	0.09	−0.014 [−0.024; −0003]	0.005	−0.143^*^	−0.006 [−0.017; 0.005]	0.006	−0.064
*F*·*R*^2^ and *R*^2^_adjusted_	*F*_(5, 981)_ = 30.67^***^; 0.135; 0.131			*F*_(5, 988)_ = 68.94^***^; 0.259; 0255			*F*_(5, 988)_ = 40.68^***^; 0.171; 0.167		
**Gender^a^**	−0.532 [−0.723; −0.34]	0.097	−0.265^***^	−0.108 [−0.266; 0.05]	0.080	−0.054	−0.272 [−0.451; −0.094]	0.091	−0.137^**^
**Age**	−0.003 [−0.011; 0.004]	0.004	−0.03	−0.026 [−0.032; −0.02]	0.003	−0.237^***^	−0.029 [−0.036; −0.022]	0.004	−0.265^***^
**Height**	0.065 [−1.03; 1.16]	0.558	0.006	1.599 [0.701; 2.497]	0.458	0.139^***^	1.13 [0.114; 2.147]	0.518	0.099^*^
Weight	−0.002 [−0.012; 0.009]	0.005	−0.021	0.003 [−0.006; 0.011]	0.004	0.034	0.001 [−0.009; 0.011]	0.005	0.012
Abdominal Perimeter	0.005 [−0.006; 0.016]	0.006	0.055	−0.005 [−0.015; 0.004]	0.005	−0.057	−0.002 [−0.012; 0.009]	0.005	−0.019
**School years**	−0.056 [−0.078; −0.035]	0.011	−0.168^***^	0.146 [0.128; 0.164]	0.010	0.436^***^	0.104 [0.084; 0.124]	0.010	0.313^***^
Former smoker^b^	−0.007 [−0.176; 0.163]	0.086	−0.003	0.202 [0.062; 0.343]	0.071	0.085^**^	0.13 [−0.029; 0.289]	0.081	0.055
Smoker^b^	0.115 [−0.131; 0.36]	0.125	0.029	−0.018 [−0.221; 0.185]	0.104	−0.005	0.011 [−0.219; 0.241]	0.117	0.003
**Alcohol 50 or less^c^**	−0.33 [−0.468; −0.192]	0.070	−0.164^***^	0.148 [0.034; 0.262]	0.058	0.074^*^	0.176 [0.047; 0.305]	0.066	0.088^**^
Alcohol more than 50^c^	−0.386 [−0.563; −0.21]	0.090	−0.165^***^	0.119 [−0.027; 0.264]	0.074	0.051	0.135 [−0.03; 0.3]	0.084	0.058
**Phy. act. less than 3^d^**	−0.153 [−0.319; 0.013]	0.085	−0.055	0.247 [0.11; 0.385]	0.070	0.088^***^	0.21 [0.054; 0.366]	0.079	0.075^**^
**Phy. act. over 3^d^**	−0.162 [−0.307; −0.016]	0.074	−0.066^*^	0.087 [−0·034;0·207]	0.061	0.035	−0.151 [−0.287; −0.014]	0.069	−0.062^*^
*F* for change in *R*^2^	*F*_(7, 974)_ = 8.63^***^			*F*_(7, 981)_ = 44.83^***^			*F*_(7, 981)_ = 19.35^***^		
*R*^2^_adjusted_; *R*^2^; Δ*R*^2^	*F*_(12, 974)_ = 18.51^***^; 0.186; 0.176; 0.051			*F*_(12, 981)_ = 63.8^***^; 0.438; 0.431; 0.180			*F*_(12, 981)_ = 30.44^***^; 0.271; 0.262; 0 101		

* p < 0.05 level;

** p < 0.01;

*** *p < 0.001*.

a *Gender, reference category: female*.

b *Reference category: nonsmoker*.

c *Alcohol measured in gr/day, reference category: none*.

d *Physical activity in number of times per week, reference category: none*.

**Figure 1:**
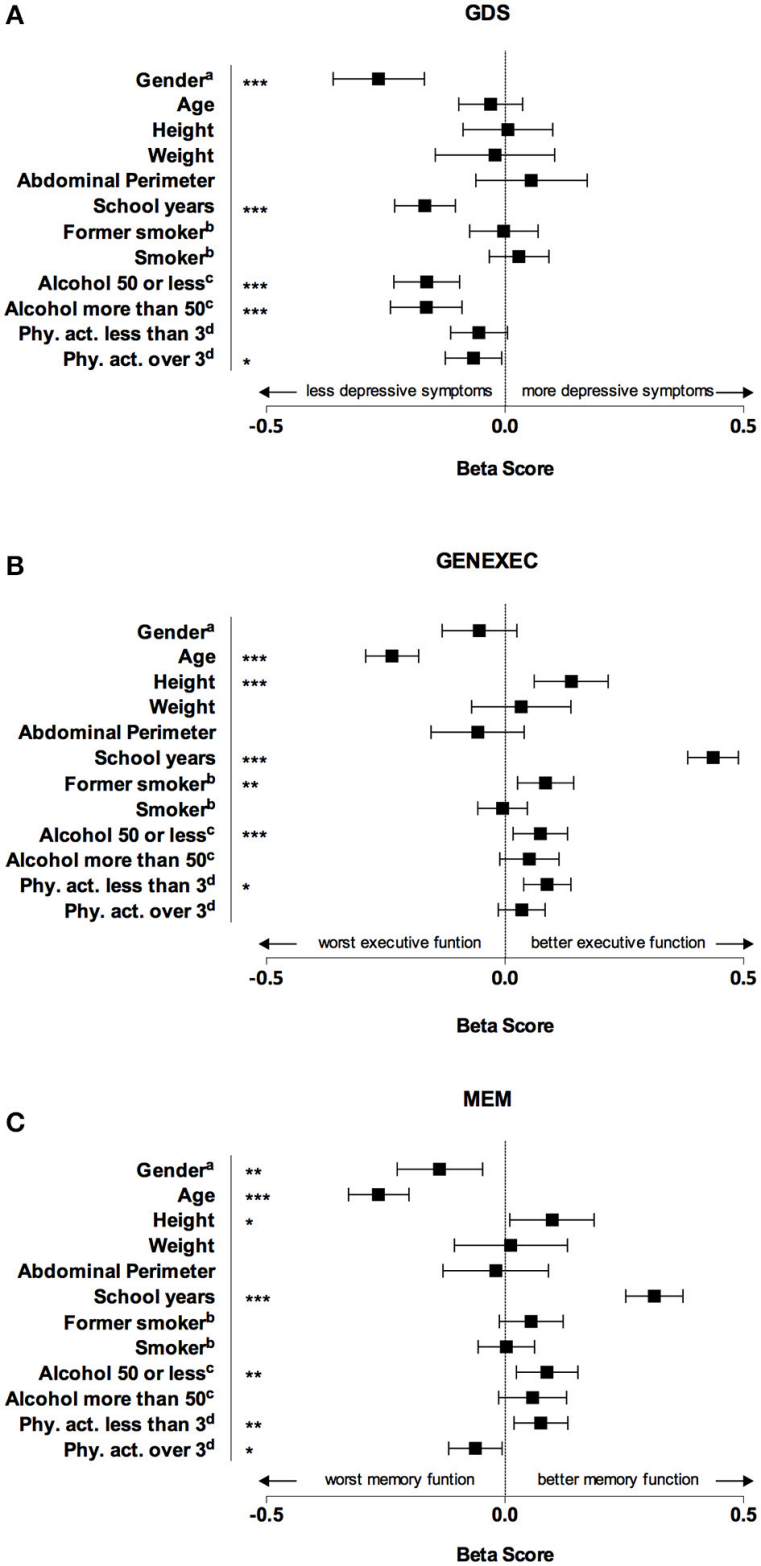
**Graphical representation of beta score (and respective confidence interval) for each dependent variable obtained in the multiple regression linear models**. Male gender, higher education, alcohol consumption, and physical activity more than 3 times a week were significantly associated with a lower score in geriatric depression scale (GDS), which means a lower prevalence of depressive symptoms **(A)**. Younger age, higher height, higher education, former smoking, moderate alcohol consumption, and physical activity were associated with a better performance in executive function score (GENEXEC) **(B)**. Female gender, younger age, higher height, higher education, moderate alcohol consumption, and physical activity were positively associated with a better performance on memory score (MEM). ^*^p < 0.05 level; ^**^p < 0.01; ^***^p < 0.001. ^a^Gender, reference category: female; ^b^Reference category: nonsmoker; ^c^measured in gr/day, reference category: none; dPhysical activity in number of times per week, reference category: none.

In the second block (Figure 1 and Table 3) variables related with the lifestyle and socio-demographic characteristics (namely school years, smoking status, alcohol consumption, and physical activity), also known to be involved in cognitive performance, were included in the model (Santos et al., 2014). The models now explained 18.6% of the variance of GDS [*F*_(12, 974)_ = 18.51; *R*^2^ = 0.186; Δ*R*^2^ = 0.051; *p* < 0.001], 43.8% of the variance of GENEXEC [*F*_(12, 981)_ = 63.8; *R*^2^ = 0.438; Δ*R*^2^ = 0.180; *p* < 0.001], and 27.1% of the variance of MEM [*F*_(12, 981)_ = 30.44; *R*^2^ = 0.271 Δ*R*^2^ = 0.101; *p* < 0.001]. Males (β = −0.265; *p* < 0.01), higher education (β = −0.168; *p* < 0.001), alcohol consumption (β = −0.164 and β = 0.165; *p* < 0.001), and physical activity more than 3 times a week (β = −0.06; *p* < 0.05) were significantly associated with a lower score in GDS. Younger age (β = −0.237; *p* < 0.001), higher height (β = 0.139; *p* < 0.001), higher education (β = 0.436; *p* < 0.001), former smoking (β = 0.085; *p* < 0.001), moderate alcohol consumption (β = 0.074; *p* < 0.05), and physical activity <3 times a week (β = 0.088, *p* < 0.001) were associated with a better performance in the composite score for general executive function. Female gender (β = −0.137; *p* < 0.001), younger age (β = −0.265; *p* < 0.01), higher height (β = 0.099, *p* < 0.05), higher education (β = 0.313; *p* < 0.001) moderate alcohol consumption (β = 0.088; *p* < 0.05), and physical activity <3 times (β = 0.075; *p* < 0.01) were positively associated with a better performance on memory tests. Adding lifestyle variables into the model showed that height is not a predictor of mood but is a significant predictor of general executive function and memory.

## Discussion

In this study, we investigated whether height and other anthropometric measures are predictors of cognitive function and mood in community-dwellers older than 50-years. Our results showed that adult body height is a good predictor of cognitive performance independently of gender, age, weight, abdominal perimeter, smoking status, alcohol consumption, and physical activity.

Compared with other studies reporting similar findings (Case and Paxson, 2008; Psaltopoulou et al., 2008; Umegaki et al., 2008; Zhang et al., 2009; Maurer, 2010; Ragonese et al., 2011; Lei et al., 2012; Guven and Lee, 2013; Quan et al., 2013; Stewart et al., 2015), our study is novel because: (1) it was conducted in a representative sample of a country that ranks within the average in OECD rankings; (2) it included a thorough evaluation of cognitive function; and (3) it considered neuropsychiatric conditions such as depressive symptoms. Many of the studies addressing the topic of height and cognition based their cognitive evaluation solely in the MMSE (Psaltopoulou et al., 2008; Yount et al., 2009; Ragonese et al., 2011; Quan et al., 2013). Although this is a widely used test to evaluate cognitive function it is not sufficient to fully characterize cognition and tends to underestimate mild cognitive impairment (Pendlebury et al., 2010). To overcome this issue, herein we performed a battery of cognitive tests and grouped them in two different dimensions: “general and executive function” and “memory” (Santos et al., 2015).

As previously shown by others, aging and educational level were the main predictors of general and executive function and memory (Santos et al., 2013, 2014; Xu et al., 2016). After aging and educational level, height was the strongest predictor of cognition and contributed significantly for the predictive value of the model. In fact, an increase in 10 cm in height was associated with an increase of 0.16 points in *z* score of GENEXEC and an increase of 0.11 points in the z score of MEM. These results show that height is an independent predictor of the different dimensions of cognitive function.

Several reasons may explain the impact of height on cognition. Both cognition and adult body height are strongly affected by early-life events and therefore adult body height may be solely the marker of an adverse childhood. From a social perspective higher height is associated with better educational attainment and social success, which may influence health by providing better social status and socio-economic environment contributing to a better cognition in adult life (Magnusson et al., 2006; Huang et al., 2015). However, while many studies did not adjust their models to school years, we show that height affects cognition independently of this parameter.

Adult body height is also affected by other factors than childhood environment. In fact, recent studies suggest that the association between height and cognition may be explained by genetic factors (Marioni et al., 2014; Joshi et al., 2015). Lower body height and cognitive function were found to have a higher chance of homozygoty. This suggests that the genetic factors influencing cognitive performance and height may be concurrent. As so, height may be just a proxy of a genetically predisposed individual for better cognitive performance (Joshi et al., 2015).

In addition, the effect of height on cognition may be the consequence of higher cognitive reserve (Santos et al., 2014). Cognitive reserve is the capacity that creates a delay in time between brain pathology and clinical expression of dementia (Singh-Manoux et al., 2011). It is known that smaller head circumference is associated with less cognitive reserve and, therefore, shorter individuals may be more prone to cognitive impairment and cognitive decline (Singh-Manoux et al., 2011). The most probable is that height influences cognition by a composite of these factors. Longitudinal studies will be of great importance to clarify this point. Interestingly, Portuguese average height is increasing in recent years; it will be interesting to analyze how the cognitive performance of our population will evolve in the years to come (Hatton, 2014).

Besides height, cognition was also associated with alcohol consumption, physical activity, and smoking status. As previously shown (Santos et al., 2014), moderate consumption of alcohol and moderate physical activity were associated with better performance in both general and executive function and memory. Of notice, gender was a significant predictor of memory (women displayed better performance than men) while it had a non-significant effect in general and executive function. Few studies had shown similar findings (Wang, 2013). Concerning smoking habits, we observed that former smokers performed better in general and executive function but not in memory. Whether these are just epiphenomena or real putative factors of cognitive impairment are questions that remain to be answered.

Another aim of our work was to explore the predictive value of weight, BMI, and abdominal perimeter in cognitive performance given that previous studies on this topic found contradictory data. While some showed an association between higher adiposity (estimated by BMI and abdominal perimeter) and worst cognitive performance, others failed to demonstrate this association (Cournot et al., 2006; Smith et al., 2014; Yesavage et al., 2014). Herein, we found that both weight (which was included instead of BMI to explore the effect of height on cognition) and abdominal perimeter had no predictive value for both general and executive function and memory when corrected for possible confounding factors. This suggests that adiposity in adulthood is not implied in cognitive performance in elderly individuals. The positive correlations between BMI and abdominal perimeter with MEM and GENEXEC are probably mediated by age (which is associated with higher abdominal perimeter) and physical activity. In spite of these observations, BMI and abdominal perimeter are only surrogate endpoints of adiposity. A more reliable marker of adiposity would be needed to clarify if this parameter is implicated in cognition and depressive symptoms.

Finally, we showed that when adjusted to sex and school years, height, weight, and abdominal perimeter are not associated with depressive symptoms. With this respect, it is interesting to highlight that male gender, school years and alcohol consumption (regardless of the quantity), were all associated with less depressive symptoms. The “protective effect” of alcohol consumption is remarkable and had already been found in different cohorts (Graham et al., 2007). In fact, it is suggested that the relation of alcohol consumption and depression may be U-shaped with both abstainers and heavy drinkers being at high risk of depression. In our study, alcohol consumption was self-reported and grouped only in two categories, which may lead to information bias; if that is the case, the observed effect is more likely to be underestimated.

The major limitation of our work is to be a cross-sectional study. A longitudinal study would be of great interest to evaluate cognitive decline instead of cognition in a specific time point. Another limitation relates with the measure of height in itself, since it is not stable but rather decreases with age. If so, the predictive effect of height in cognition may be overestimated since older individuals are smaller (as can be observed in Table 2). However, the multiple regression models included age and, even so, the predictive value of height remained significant. A good option for further studies would be to include the measure of foot-to-knee since it is more constant after puberty.

In spite of these limitations the data support the conclusion that height is a predictor of cognitive function but not depressive symptoms in the elderly. Weight and abdominal perimeter do not associate with any of these parameters. Longitudinal studies are expected to bring more light into this subject.

## Author contributions

VP, NCS, and PC performed the statistical analysis. VP, NCS, PC, MC, JP, and NS contributed to the data analyses and discussion. NCS maintains the database, organized the neurocognitive/psychological sessions and collected the data. PGC organized the evaluation sessions, and participant recruitment and collected the data. VP wrote the first draft of the manuscript. NS and JP conceived and designed the study. All the authors revised the manuscript. NS had access to all the data in the study.

## Funding

This work was funded by the European Commission (FP7) “SwitchBox” (Contract HEALTH-F2-2010-259772) project and co-financed by the Portuguese North Regional Operational Program (ON.2—O Novo Norte) under the National Strategic Reference Framework (QREN), through the European Regional Development Fund (FEDER), and by Fundação Calouste Gulbenkian—Inovar em Saúde (“Envelhecimento cognitivo saudável—proporcionar saúde mental no processo biológico do envelhecimento,” Contract P-139977). NCS is supported by a SwitchBox post-doctoral fellowship.

### Conflict of interest statement

The authors declare that the research was conducted in the absence of any commercial or financial relationships that could be construed as a potential conflict of interest.
